# Subacute Exposure to an Environmentally Relevant Dose of Di-(2-ethylhexyl) Phthalate during Gestation Alters the Cecal Microbiome, but Not Pregnancy Outcomes in Mice

**DOI:** 10.3390/toxics9090215

**Published:** 2021-09-09

**Authors:** Karen Chiu, Shah Tauseef Bashir, Liying Gao, Jessica Gutierrez, Maria R. C. de Godoy, Jenny Drnevich, Christopher J. Fields, Isaac Cann, Jodi A. Flaws, Romana A. Nowak

**Affiliations:** 1 Division of Nutritional Sciences, College of Agricultural, Consumer and Environmental Sciences, University of Illinois, Urbana, IL 61802, USA; kkchiu2@illinois.edu (K.C.); mgodoy2@illinois.edu (M.R.C.d.G.); 2 Department of Comparative Biosciences, College of Veterinary Medicine, University of Illinois, Urbana, IL 61802, USA; lgao@illinois.edu (L.G.); gutierrezjessica042@gmail.com (J.G.); 3 Department of Molecular and Integrative Physiology, College of Liberal Arts & Sciences, University of Illinois, Urbana, IL 61802, USA; tbshah3@illinois.edu; 4 Department of Animal Sciences, College of Agricultural, Consumer and Environmental Sciences, University of Illinois, Urbana, IL 61802, USA; icann@illinois.edu (I.C.); ranowak@illinois.edu (R.A.N.); 5 Carl R. Woese Institute of Biology, University of Illinois, Urbana, IL 61802, USA; 6 High Performance Computing in Biology, Roy J. Carver Biotechnology Center, University of Illinois, Urbana, IL 61802, USA; drnevich@illinois.edu (J.D.); cfields@illinois.edu (C.J.F.); 7 Department of Microbiology, College of Liberal Arts & Sciences, University of Illinois, Urbana, IL 61802, USA

**Keywords:** endocrine-disrupting chemicals, di-(2-ethylhexyl) phthalate (DEHP), gut microbiome, gestational exposure

## Abstract

Di-2-ethylhexyl phthalate (DEHP) is a plasticizer commonly found in polyvinyl chloride, medical equipment, and food packaging. DEHP has been shown to target the reproductive system and alter the gut microbiome in humans and experimental animals. However, very little is known about the impact of DEHP-induced microbiome changes and its effects during pregnancy. Thus, the objective of this study was to investigate the effects of DEHP exposure during pregnancy on the cecal microbiome and pregnancy outcomes. Specifically, this study tested the hypothesis that subacute exposure to DEHP during pregnancy alters the cecal microbiome in pregnant mice, leading to changes in birth outcomes. To test this hypothesis, pregnant dams were orally exposed to corn oil vehicle or 20 µg/kg/day DEHP for 10 days and euthanized 21 days after their last dose. Cecal contents were collected for 16S Illumina and shotgun metagenomic sequencing. Fertility studies were also conducted to examine whether DEHP exposure impacted birth outcomes. Subacute exposure to environmentally relevant doses of DEHP in pregnant dams significantly increased alpha diversity and significantly altered beta diversity. Furthermore, DEHP exposure during pregnancy significantly increased the relative abundance of Bacteroidetes and decreased the relative abundance of Firmicutes and Deferribacteres compared with controls. The affected taxonomic families included *Deferribacteraceae*, *Lachnospiraceae*, and *Mucisprillum*. In addition to changes in the gut microbiota, DEHP exposure significantly altered 14 functional pathways compared with the control. Finally, DEHP exposure did not significantly impact the fertility and birth outcomes compared with the control. Collectively, these data indicate that DEHP exposure during pregnancy shifts the cecal microbiome, but the shifts do not impact fertility and birth outcomes.

## 1. Introduction

Phthalates are plasticizers commonly added to polyvinyl chloride to create soft and malleable plastics. These chemicals are stabilizers for many solvents to retain color and fragrance. Phthalates can be found in children’s toys, clothing (faux leather or printed vinyl materials on clothes), personal care products (nail polish, lotion, deodorants, perfumes, and shampoo), medical equipment (blood bags and tubing), and housing materials (vinyl flooring, carpets, and shower curtains). Di-2-ethylhexyl phthalate (DEHP) is one of the most abundant phthalates, and it is detectable in human blood, urine, amniotic fluid, and breastmilk samples [1,2,3]. DEHP is not chemically bound to products. Thus, repeated washing, exposure to heat/light, and changes in pH will cause DEHP to leach out of the materials and into the environment. As a result, humans are exposed to DEHP through ingestion, inhalation, and dermal absorption, with ingestion being the most common route of exposure. Intravenous absorption of DEHP is another common route of exposure in specific populations, such as patients on dialysis, tube feedings, or intravenous infusions [4,5].

Several epidemiological studies reported that DEHP exposure affects pregnant women and birth outcomes [6,7,8]. Specifically, women at the 75th percentile of DEHP metabolite concentrations have a longer gestational period than women at the 25th percentile [7]. DEHP metabolites were also associated with increased odds of delivering at 41 weeks or later and delivering through Cesarean section [7]. Furthermore, animal studies have strengthened this finding by indicating that DEHP exposure also impacts birth outcomes in rodents [9]. For example, gestational exposure to DEHP significantly impaired embryo implantation, increased embryo loss, and reduced endometrial receptivity compared with the control in mice [9]. Maternal exposure to DEHP also interfered with the normal development of the placenta by disrupting placental growth and placental vascularization in mice. Prenatal exposure to DEHP also increased the male-to-female sex ratio in the F1 generation, increased time to pregnancy, and caused a loss of some pups in mice [10]. These data indicate that DEHP exposure severely impacts pregnancy and birth outcomes.

In addition to affecting pregnancy and birth outcomes, DEHP exposure has been shown to impact the gut microbiome in nonpregnant animals [11,12,13]. Further, review articles have also discussed the maternal gut microbiome, toxicants, and its associated with pregnancy outcomes [14,15]. Specifically, bacterial species that have been identified and associated with spontaneous preterm birth include *Mycoplasma hominis*, *Fusobacterium nucleatum*, and *Bacteroides* spp. Although DEHP exposure is linked to both adverse pregnancy outcomes and changes in the nonpregnant microbiome, studies have not investigated the impact of DEHP exposure during pregnancy on the gut microbiome. Microbial composition is different in each segment of the gastrointestinal tract, with the lowest density and bacterial diversity in the stomach and the highest density and bacterial diversity in the colon [16]. The colon consists of the cecum (the beginning of the colon), ascending colon (first part of the colon), transverse colon (second part of the colon), descending colon (third part of the colon), and sigmoid colon (last part of the colon). Because the colon is the most diverse segment along the gastrointestinal tract, this study focused on characterizing the effects of DEHP exposure during pregnancy on microbial and functional changes in the colon, specifically the cecum. Further, this study focused on the effects of DEHP exposure during pregnancy on the cecal microbiome using 16S rRNA gene sequencing for bacterial identification and shotgun metagenomic sequencing for determining the microbiome’s functional potential. Finally, this study explored whether DEHP-induced shifts in the microbiome were associated with DEHP-induced pregnancy and birth outcomes. Specifically, this study tested the hypothesis that exposure to DEHP during pregnancy modifies the cecal microbiome in pregnant dams, leading to changes in birth outcomes.

## 2. Materials and Methods

### 2.1. Chemicals

DEHP and tocopherol-stripped corn oil were used in these studies. DEHP was purchased from Sigma-Aldrich (St. Louis, MO, USA), and the tocopherol-stripped corn oil was purchased from MP Biomedicals, LLC. The 20 µg/kg DEHP dose was created by serially diluting the DEHP stock (as purchased) with corn oil. This dose of 20 µg/kg DEHP is an environmentally relevant dose because it falls within the average dose of human exposure to DEHP, which is 1–30 µg/kg/day [17]. Corn oil was used as vehicle control.

### 2.2. Experimental Animals

This experiment was conducted in an AALAC (Association for Assessment and Accreditation of Laboratory Animal Care)-approved animal facility located at the University of Illinois at Urbana-Champaign Veterinary Medicine Basic Science Building. Male and female CD-1 mice were purchased from Charles River (Wilmington, MA, USA) and were allowed to acclimate to the new facility for two weeks. The facility that housed these mice maintained ambient temperatures at 21.1 ± 2.2 °C with humidity at 50 ± 20%. Mice were kept on 12/12 h (light/dark) cycles. Mice were also given ad libitum access to Tekland Rodent Diet (8604) and reverse-osmosis-treated water. All animal procedures were approved by the University of Illinois Institutional Animal Care and Use Committee (Protocol No.: 17079).

### 2.3. Experimental Design

**Figure 1:**
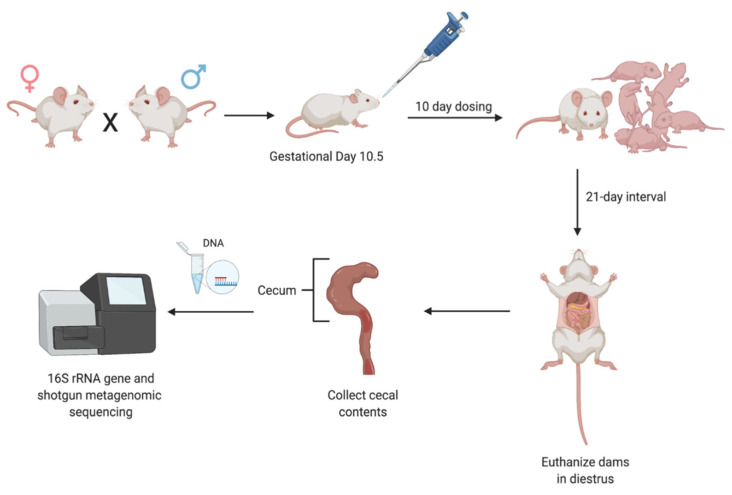
Experimental timeline of DEHP dosing in dams. Briefly, adult male and female mice were bred to generate pregnant mice. Pregnant mice were orally dosed with corn oil (vehicle control) or DEHP for 10 days. Dams were euthanized 21 days after their last dose. Cecal contents were collected from the dams for 16S rRNA gene and shotgun metagenomic sequencing. Pups were monitored for their birth outcomes.

At 2 months of age (60 days of age), female mice (*n* = 6 in control group, *n* = 6 in DEHP group) were bred with male mice. Once the female mice were pregnant, they were separated from the male mice and housed individually. Then, pregnant mice were orally dosed by gently pipetting either vehicle control (corn oil) or 20 µg/kg DEHP starting at gestational day (GD) 10.5 for 10 days until the dams gave birth. After 10 days of dosing with either the control vehicle (corn oil) or DEHP, dams in diestrus were euthanized 21 days after their last dose by CO_2_ asphyxiation and cervical dislocation. See Figure 1 for a summary of the experimental timeline.

### 2.4. Cecal Collection

Following euthanization, cecal digesta were collected for microbiome sequencing because the colon, which includes the cecum, cultivates the most diverse and dense communities compared with all other body habitats [18]. Cecal digesta were collected within 15 min of death and placed into sterile cryovials. The cryovials were then snap-frozen in liquid nitrogen immediately and stored at −80 °C until DNA extraction for 16S rRNA gene and shotgun metagenomic sequencing.

### 2.5. DNA Isolation and Quantification

Total bacterial DNA was extracted from cecal digesta samples using Mo-Bio PowerSoil kits (MO BIO Laboratories, Inc., Carlsbad, CA, USA). The protocol for DNA extraction was followed according to the manufacturer’s guidelines and instructions. Briefly, cecal contents were added to PowerBead tubes (which contain beads) for rapid and thorough mechanical homogenization. Following cell lysis, the mixture was transferred to a spin column tube to capture genomic DNA on a silica membrane. Finally, DNA was washed and eluted from the membrane.

Following DNA extraction, microbial genomic DNA concentration was quantified using a Qubit^®^ 2.0 Fluorometer (Life technologies, Grand Island, NY, USA), and DNA quality was confirmed by running the DNA samples on a 1% agarose gel. All samples had no smearing and had a molecular weight greater than 10 kb. No samples showed signs of RNA contamination, DNA degradation, or impurities, thus all samples were included for 16S rRNA gene and shotgun metagenomic sequencing.

### 2.6. Processing of Microbial Genomic DNA for 16S rRNA Gene and Metagenomic Sequencing

Both 16S rRNA gene Illumina and shotgun metagenomic sequencing were performed at the Roy J. Carver Biotechnology Center at the University of Illinois. Fluidigm Access Array (Fluidigm Corporation, South San Francisco, CA, USA) in combination with Roche High Fidelity Fast Start Kit (Roche, Indianapolis, IN, USA) was used to generate 16S rRNA gene amplicons. The same DNA samples were also used for shotgun genomic libraries, prepared using the Hyper Library construction kit from Kapa Biosystems (Roche, Indianapolis, IN, USA).

For the 16S rRNA gene, the primers 515F (5′-GTGCCAGCMGCCGCGGTAA-3′) and 806R (5′-GGACTACHVHHHTWTCTAAT-3′) that target a 291 bp-fragment of V4 region were used for amplification (primers synthesized by IDT Corp., Coralville, IA, USA). CS1 forward tag (5’-ACACTGACGACATGGTTCTACA-3′) and CS2 reverse tag (5’-TACGGTAGCAGAGACTTGGTCT-3′) were added according to the Fluidigm protocol. Fragment Analyzer (Advanced Analytics, Ames, IA, USA) confirmed the quality of amplicons as well as their regions and sizes. A DNA pool was generated by combining equimolar amounts of the amplicons from each sample. Then, the pooled samples were size selected on a 2% agarose E-gel (Life technologies, Grand Island, NY, USA) and extracted using a Qiagen gel purification kit (Qiagen, Valencia, CA, USA). Samples and primers (4 µL each) were loaded into their respective sample and primer inlets of a primed Fluidigm 48.48 Access Array integrated fluidic circuit (IFC) and placed in the Juno microfluidic machine (Fluidigm Corporation). Polymerase chain reaction (PCR) cycling conditions were followed according to Fluidigm Illumina Sequencing Systems. Briefly, samples were loaded in 384-well plates into the PCR thermal cycler. Next, 35 cycles of PCR were run: 50 °C 2 min, 70 °C 20 min, 95 °C 10 min (1 cycle); 95 °C 15 s, 60 °C 30 s, 72 °C 1 min (10 cycles); 95 °C 15 s, 80 °C 30 s, 60 °C 30 s, 72 °C 1 min (2 cycles); 95 °C 15 s, 60 °C 30 s, 72 °C 1 min (8 cycles); 95 °C 15 s, 80 °C 30 s, 60 °C 30 s, 72 °C 1 min (2 cycles); 95 °C 15 s, 60 °C 30 s, 72 °C 1 min (8 cycles); and 95 °C 15 s, 80 °C 30 s, 60 °C 30 s, 72 °C 1 min (5 cycles). Cleaned size-selected pooled products were run on an Agilent Bioanalyzer to confirm the appropriate profile and average size. Illumina sequencing was performed on a MiSeq using v3 reagents (Illumina Inc., San Diego, CA, USA).

For the preparation of shotgun libraries, DNA fragments had an average size of 280 bp as determined by tracing the Bioanalyzer library. The libraries were quantitated by quantitative polymerase chain reaction (qPCR) and sequenced on two lanes for 161 cycles from one end of the fragments on a HiSeq 2500 using a HiSeq SBS sequencing kit version 4. Fastq files were generated and demultiplexed with the bcl2fastq v2.17.1.14 Conversion Software (Illumina Inc., San Diego, CA, USA). Illumina library adapter sequences were trimmed from the 3’-end of R1 (AGATCGGAAGAGCACACGTCTGAACTCCAGTCAC) and R2 (AGATCGGAAGAGCGTCGTGTAGGGAAAGAGTGT). Over 1.2 billion reads were produced.

### 2.7. Bioinformatic Analyses of 16S rRNA Gene Data

The 16S rRNA gene data analyses were completed by the High-Performance Computing in Biology (HPCBio) group at the University of Illinois. The Fluidigm data targeting the V4 region of the 16S rRNA gene were processed using the TADA Nextflow-based workflow (https://github.com/h3abionet/TADA) that implements the DADA2 workflow [19] for dereplicating and denoising reads to generate amplicon sequence variants (ASVs). In brief, primer sequences were removed, and reads were truncated to 240 nt in length. Sequences with unclassified bases, i.e., with Ns instead of the normal nucleotides, were removed. Default steps were used to denoise reads, merge paired reads into a single amplicon sequence, and dereplicate into ASVs, followed by bacterial taxonomic assignment using the DADA2 implementation of the RDP Classifier [20] and the Silva v132 database [21]. Multiple sequence alignment of the resulting ASV sequences was performed by DECIPHER followed by a midpoint-rooted Fasttree [22] phylogenetic analysis to produce a maximum likelihood tree used in data analysis steps. Raw counts, taxonomic assignments, and the phylogenetic tree for the ASVs were imported into R v. 4.0.3 (R Core Team 2020) using the package phyloseq v. 1.34.0 (McMurdie and Holmes 2013). One replicate in the DEHP treatment group had substantially fewer total raw counts and fewer ASVs compared with all the other samples, and was thus discarded, leaving *n* = 5 replicates for the DEHP 16S rRNA gene data analysis. ASVs were filtered out if they were mitochondrial or chloroplast, did not have an assigned phylum, or were the only ASV in a phylum. ASVs were agglomerated if they had extremely close phylogenetic distances <10^−2.5^, adding their counts together, leaving 221 “taxa” for statistical analysis of alpha diversity (Observed species richness, Chao1 and FaithPD estimates) and beta diversity (Bray–Curtis, UniFrac and Weighted UniFrac estimates). Taxa were further agglomerated to Phylum, Family, and Genus levels and differences in relative abundances of the top 10 groups were determined between control and DEHP using *t*-tests.

In this study, multiple measurements of alpha and beta diversity were reported because each of these indices yield slightly different, but valuable information on alpha and beta diversity. Alpha diversity measures the richness and evenness of a given sample, and the most commonly used indices for measuring alpha diversity include Observed, Chao1, and FaithPD. Observed and Chao1 indices do not factor in the phylogeny of the taxa identified during sequencing, whereas FaithPD uses phylogenetic distance to calculate the diversity in a particular sample [23]. Chao1 and Observed are similar in that they do not take phylogenetic diversity into account; however, Chao1 gives more weight to low abundance or rare species compared with Observed [23]. Beta diversity measures the difference between two or more treatment groups. Common indices for beta diversity include Bray–Curtis, UniFrac, and weighted UniFrac. All three of these indices were included because each index also yields slightly different, yet valuable information. The Bray–Curtis dissimilarity is a measurement that incorporates the phylogenetic distances between the observed organisms. Weighted UniFrac is a variation of the unweighted UniFrac where it accounts for relative abundances [24].

### 2.8. Bioinformatic Analyses of Metagenomic Data

Briefly, sequence data were processed using a Nextflow-based workflow to maintain consistency and reproducibility [25]. Initial data were quality-assessed using FASTQC [26] and trimmed using Trimmomatic v. 0.36 [27]. Trimmed paired-end reads were then combined using VSEARCH v. 2.8.0 [28], retaining the first read from the pair if the reads did not overlap. Sequences originating from the host were filtered from the joined reads using kneadData v. 0.6.15 [29]. Reads were aligned to the NCBI nr protein database (June 2018 release) using DIAMOND v. 0.9.22 [30], retaining the top five hits. Alignment results were annotated using MEGAN v. 6.12.0 Ultimate Edition [31] using a customized mapping file generated on the June 2018 NCBI nr release and from publicly available MEGAN functional mapping files obtained from the same month. Raw count data from taxonomic (NCBI taxonomy) and functional (KEGG) classification results per node were exported from MEGAN in BIOM format. These were analyzed using R v. 3.5.2 [32] and RStudio in R markdown format, with initial data importing and primary data analysis data utilizing the R package phyloseq v. 1.26 [33]. Alpha diversity analyses were performed using the R packages phyloseq and vegan v. 2.5 [34]; a standard R-based *t*-test was performed to assess differences between control and treatment effects. Beta diversity analyses were performed in R using phyloseq and vegan using normalized sample counts via the R package metagenomeSeq v. 1.24 [35]. PERMANOVA and ANOSIM tests were performed using vegan functions ‘adonis2′ and ‘anosim’ to assess significance of differences in Bray–Curtis distance between control and treatment. Differential abundance analysis was performed using normalization methods developed for phyloseq using DESeq2 [36]. Simple KEGG feature enrichment analysis was performed using the function ‘kegga’ from the R package limma [37] using KEGG orthology IDs showing differential abundance with an adjusted *p*-value cutoff of ≤0.1. Random forest analysis was performed on taxonomic and functional features using the R package randomForest v. 4.16 [38] to assess the rank importance of features that classify samples into control and treatment conditions.

### 2.9. Pregnancy Outcomes

Breeding studies were conducted by placing two adult CD-1 female mice with one adult CD-1 male mouse (7–8 weeks old) per cage. Copulatory plugs were checked every morning for a maximum of 14 days. Female mice with visible copulatory plugs were placed in a new, clean cage. The weights of each mouse were recorded twice per week to monitor and ensure these mice were gaining weight as a result of pregnancy. A weight gain of 4 g or more ensured that mice were gaining proper weight for the duration of their pregnancy. Fertility was monitored by conducting fertility tests and examining the gestational index. The gestational index was calculated by the number of females who gave birth to pups divided by the number of pregnant females, and then multiplying the number by 100. Gestational index is reported as a percentage. Once the female dams gave birth to pups, all pups were counted and sexed. Then, the live and dead pups were sexed, separated, and recorded. The pup litter weights were recorded from only the live pups. The average weight of the pups was determined by taking the litter weight divided by the number of live total pups. In addition to collecting weights of the dams and pups, organ weights (i.e., liver, spleen, uterus, and ovary) were also measured in the dams. When the pups reached the age of postnatal day (PND) 21, the anogenital distance (AGD) was measured and body weight was recorded to determine the anogenital index (AGI). The AGI was calculated by taking the AGD divided by the cubed root of body weight.

### 2.10. Statistical Analysis

The body and organ weight data were analyzed using *t*-tests and Mann–Whitney tests on GraphPad Prism version 8.4.3 for MacOS. Shapiro–Wilk tests were used to test data sets for normality. Data that were normally distributed (*p* > 0.05) were assessed for statistical significance by unpaired *t*-test. The parametric *t*-test was used for determining whether body weight, liver, spleen, uterus, and ovary weights were statistically significant. The data on anogenital distance (AGD) were not normally distributed (*p* < 0.05), so statistical differences for this end-point were assessed by Mann–Whitney tests.

## 3. Results

### 3.1. Cecal Microbiota

#### 3.1.1. Alpha and Beta Diversity

**Figure 2:**
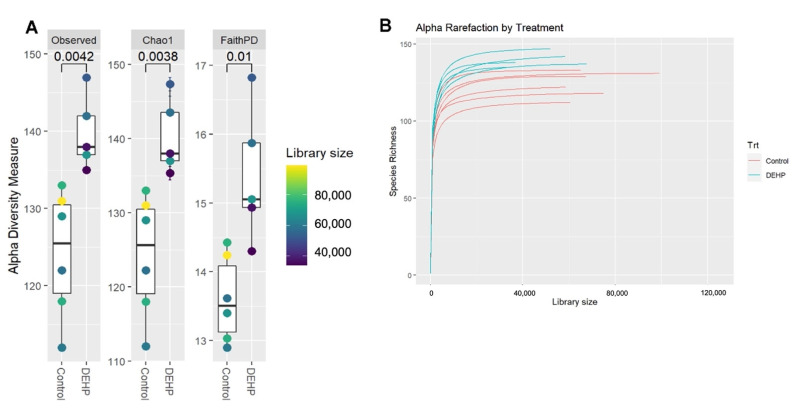
Alpha diversity of pregnant mice treated with control or DEHP. Alpha diversity metrics used in the current study included Observed, Chao1, and FaithPD after agglomeration and filtering (**A**) and rarefaction (**B**). *p*-values from Student’s *t*-test are indicated in panel **A**. Statistical significance is defined at *p* < 0.05. *n* = 5–6/treatment group.

**Figure 3:**
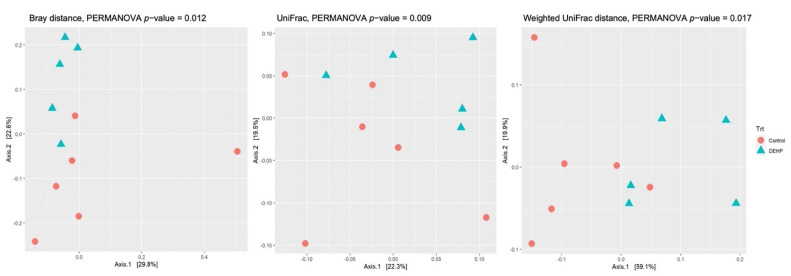
Beta diversity of pregnant mice treated with control or DEHP. PCoA plots of three beta diversity metrics (Bray distance, unweighted UniFrac distance, and weighted UniFrac distance) revealed distinct separation between control and DEHP treatment groups (*p* < 0.05). Orange circles indicate control group, and blue triangles indicate DEHP treatment group. PERMANOVA *p*-values are indicated above the PCoA plots, and statistical significance is defined at *p* < 0.05. *n* = 5–6/treatment group.

After agglomeration and filtering, measurements of cecal bacterial alpha diversity indicated that DEHP-exposed mice had significantly greater species richness compared with control mice as measured by Observed (*p* = 0.0042), Chao1 (*p* = 0.0038), and FaithPD (*p* = 0.01) (Figure 2). The alpha diversity measures the diversity or variation of microbes in one sample. Several alpha-diversity metrics were used in the current study to determine the richness and distribution within a sample. Beta-diversity metrics using Bray distance (*p* = 0.012), unweighted UniFrac (*p* = 0.009), and weighted UniFrac (*p* = 0.017) showed that DEHP exposed dams were significantly clustered together and away from control dams (Figure 3). Unlike alpha diversity, beta diversity measures the similarity or dissimilarity between two or more samples or communities. Several metrics were also used in the current study to measure beta diversity, and each metric examined different aspects, such as size or shape, of the communities.

#### 3.1.2. Taxonomic Results

**Figure 4:**
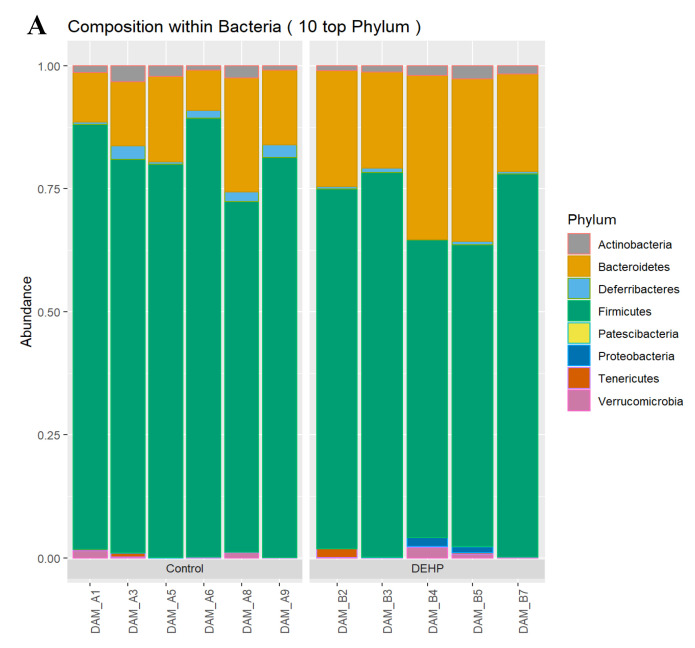

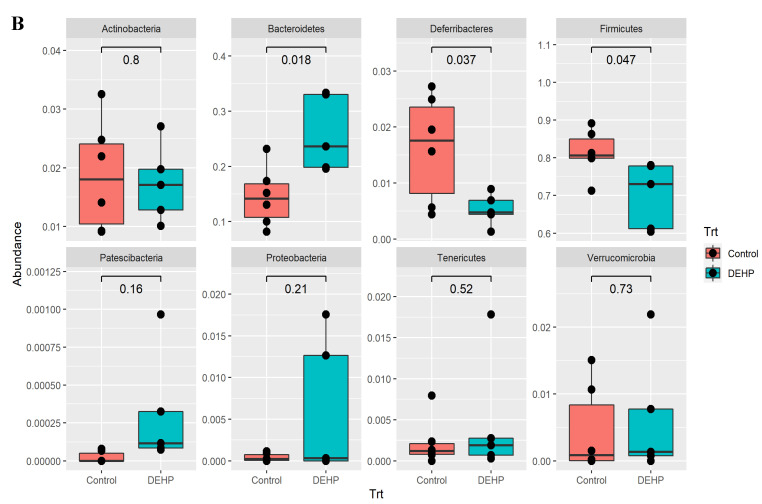
Overall relative abundance composition of cecal bacteria for the eight phyla that were identified in each sample (**A**). The relative abundance of each phyla by treatment group is shown in (**B**). *n*= 5–6/treatment group.

Taxonomic analysis from the 16S (V4) rRNA genes data indicated that the most abundant phyla were Firmicutes and Bacteroidetes, which accounted for over 90% of all sequences. Actinobacteria, Deferribacteres, Patescibacteria, Proteobacteria, Tenericutes, and Verrucomicrobia were the additional bacterial phyla identified in our samples, which comprised the remaining percentage of the sequences (Figure 4). DEHP exposure significantly altered three bacterial phyla. Specifically, subacute exposure to DEHP significantly increased the relative abundance of Bacteroidetes compared with the control (*p* = 0.018). DEHP exposure also significantly decreased the relative abundance of Deferribacteres (*p* = 0.037) and Firmicutes (*p* = 0.047) compared with the control. The remaining phyla analyzed, including Actinobacteria, Patescibacteria, Proteobacteria, Tenericutes, and Verrucomicrobia, were not significantly changed in the different treatment groups (*p* > 0.05).

**Figure 5:**
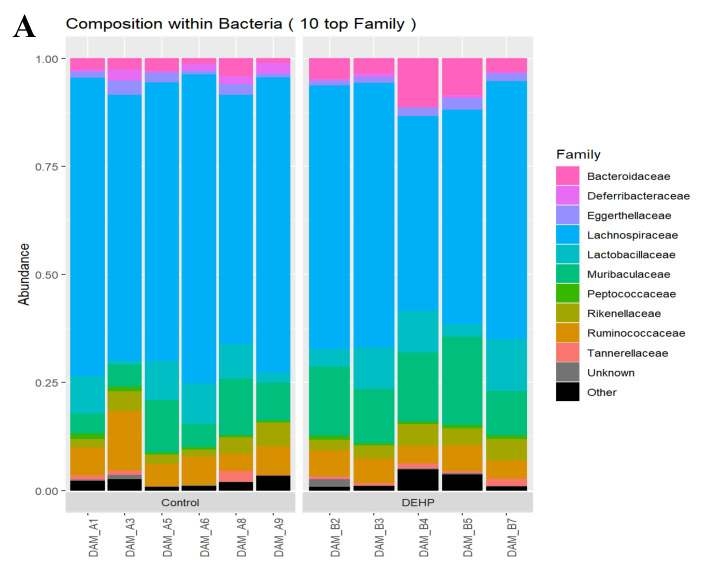

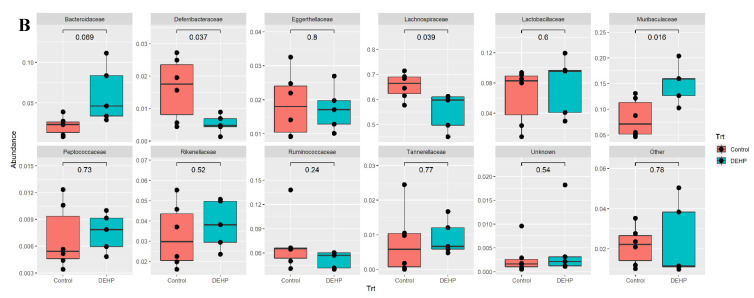
Overall family composition of samples in dams (**A**) and relative abundance of each family by treatment group (**B**). *n* = 5–6/treatment group.

The relative abundance composition for the top 10 families included Bacteroidaceae, Deferribacteraceae, Eggerthellaceae, Lachnospiraceae, Lactobacillaceae, Muribaculaceae, Peptococcaceae, Rikenellaceae, Ruminococcaceae, and Tannerellaceae (Figure 5). Subacute exposure to DEHP significantly decreased the relative abundance of Deferribacteraceae (*p* = 0.037) and Lachnospiraceae (*p* = 0.039). In addition to changes in Deferribacteraceae and Lachnospiraceae, subacute exposure to DEHP significantly increased the relative abundance of Muribaculaceae (*p* = 0.016) compared with the control. Finally, the relative abundance of Bacteroidaceae was borderline increased in dams exposed to DEHP compared with the control (*p* = 0.069).

**Figure 6:**
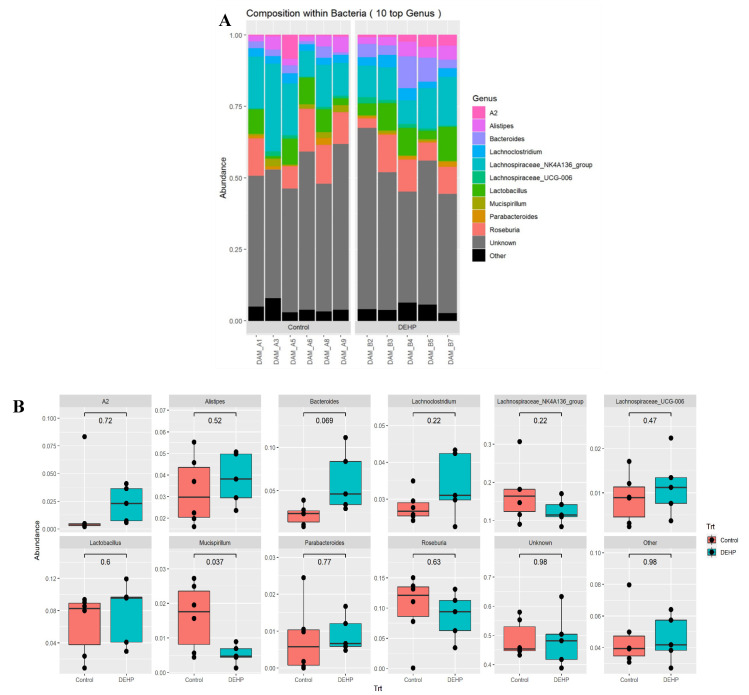
Overall genus composition of samples in dams (**A**) and relative abundance of each genus by treatment group (**B**). *n* = 5–6/treatment group.

For taxonomy at the genus level, dams exposed to DEHP had a significant reduction in the relative abundance of *Mucisprillum* compared with the control (*p* = 0.037, Figure 6B). The relative abundance of Bacteroides in DEHP treated borderline increased compared with the control (*p* = 0.069). Finally, the other genus analyzed, including *A2*, *Alistipes*, *Lachnoclostridium*, *Lachnospiraceae NK4A136*, *Lactobacillus*, *Parabacteroides*, and *Roseburia*, were not statistically different compared with the control (Figure 6B). The number of taxa with assigned ranks at each level can be found in Table 1. 

To assess the effects on the association of treatment with beta diversity values, a model of Trt + one covariate for the significant and marginal covariates was analyzed using PERMANOVA. In all models, adding the covariate led to decreased *p*-values for the association of treatment with beta diversity values, indicating that the significant/marginal associations of the covariates slightly obscured the effect of treatment, rather than artificially increasing it (Table 2).

### 3.2. Cecal Microbiome

#### 3.2.1. Alpha and Beta Diversity

**Figure 7:**
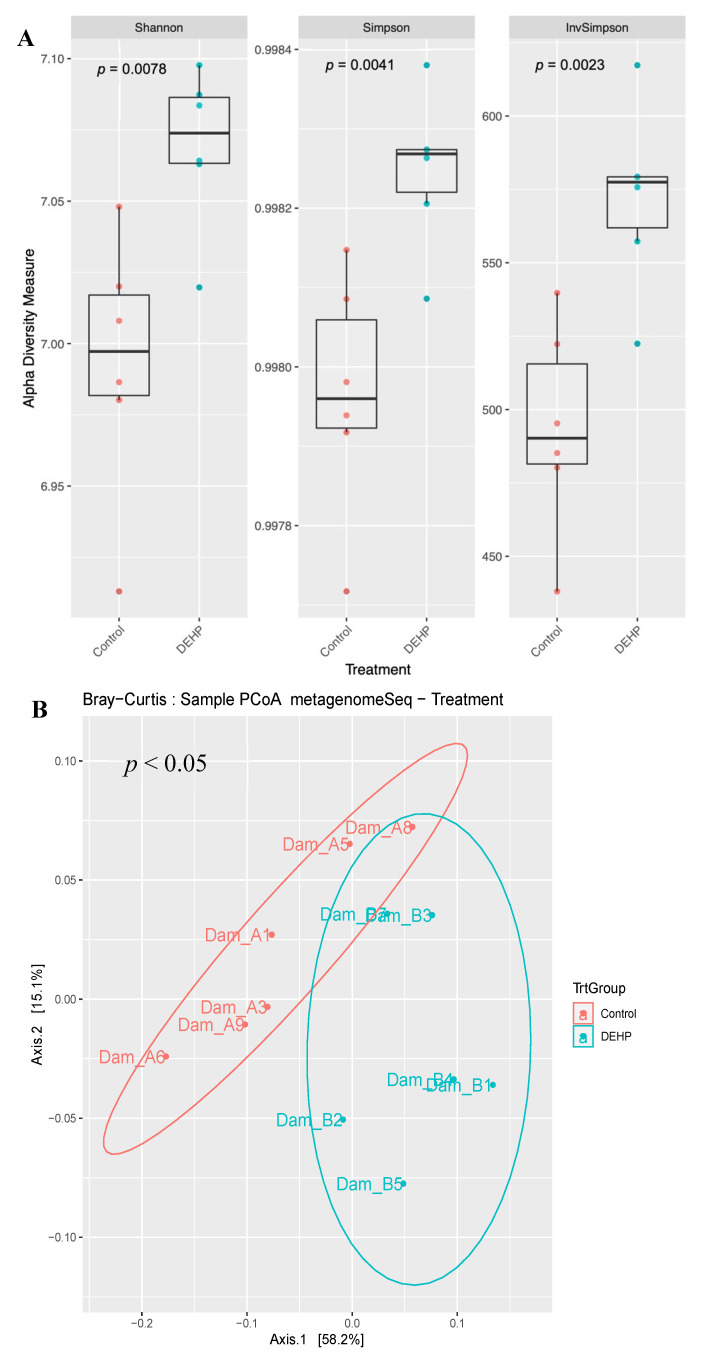
Shannon, Simpson, and inverse Simpson alpha diversity of functional cecal metagenome of control and DEHP exposed dams (**A**). Bray–Curtis beta diversity of functional analysis of control and DEHP exposed dams (**B**).

Three different alpha diversity metrics were used (Shannon, Simpsons, and Inverse Simpson), and all of these alpha diversity metrics showed greater species richness in the DEHP exposed treatment group compared with the control (*p* < 0.05, Figure 7A). Furthermore, the PCoA plot showed that DEHP exposed dams are clustered together and away from control dams (*p* < 0.05, Figure 7B).

#### 3.2.2. Results from Functional Analysis

The metabolic potentials of DEHP-exposed dams were compared to control dams by shotgun metagenomic sequencing. Specifically, 14 functional profiles were significantly altered in DEHP-exposed dams compared with control dams (*p* < 0.05). Functional pathways that were significantly altered include flagellar assembly, bacterial chemotaxis, protein export, oxidative phosphorylation, carbon fixation pathways in prokaryotes, quorum sensing, homologous recombination, ubiquinone and other terpenoid-quinone biosynthesis, histidine metabolism, secondary bile acid biosynthesis, vancomycin resistance, carbon metabolism, and citrate acid cycle (Table 3). Other functional pathways (i.e., mismatch repair, alanine/aspartate/glutamate metabolism, biotin metabolism, apoptosis, and carbapenem biosynthesis) were examined, but were marginally different between treatment groups (Table 3, *p* < 0.10).

#### 3.2.3. Gestational Outcomes and Weights in Dams

**Figure 8:**
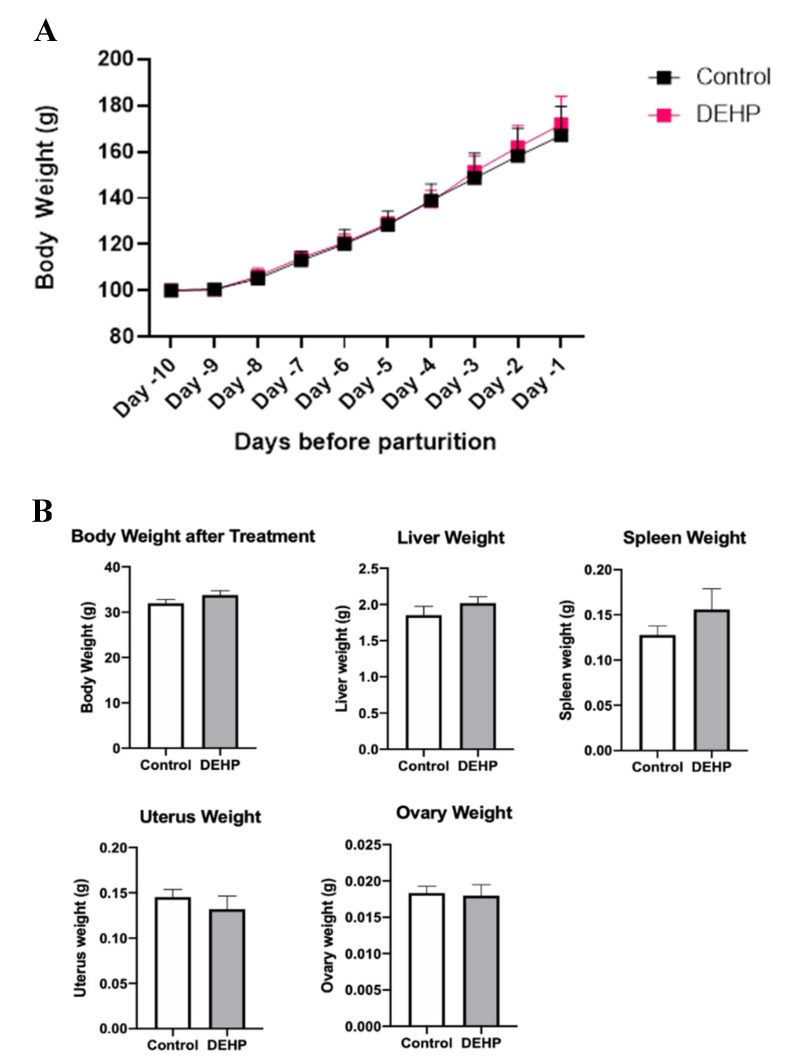
Body (**A**) and organ weights (**B**) of control and DEHP-treated dams. The data are presented as mean ± standard error of the mean (SEM), *n* = 6/treatment group.

**Figure 9:**
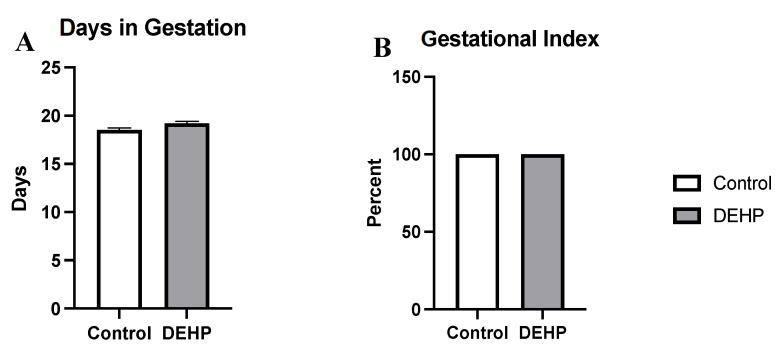
Days in gestation (**A**) and gestational index (**B**) in pregnant mice. The data in panel **A** are presented as mean ± standard error of the mean (SEM), *n* = 6/treatment group.

DEHP exposure during pregnancy did not significantly alter body weight in female dams compared with the control (*p* = 0.186, Figure 8A). DEHP exposure during pregnancy also did not significantly alter liver weight (*p* = 0.296), spleen weight (*p* = 0.281), uterine weight (*p* = 0.447), and ovary weight (*p* = 0.839) in the dams compared with the control, as shown in Figure 8B. Further, DEHP exposure during pregnancy did not significantly affect the gestational index compared with the control (Figure 9).

Permutational multivariate analysis of variance (PERMANOVA) was implemented to determine if treatment and/or tissue weight covariates were significantly associated with the distance matrices generated in Table 2. Treatment alone was significant for Bray–Curtis dissimilarity (*p* = 0.012), UniFrac (*p* = 0.009), and weighted UniFrac distance (*p* = 0.017). Liver weight alone was also significant for weighted UniFrac distance (*p* = 0.046). Uterine, spleen, and body weights were marginally associated with UniFrac distance (*p* = 0.057), weighted UniFrac (*p* = 0.072), and weighted UniFrac (*p* = 0.082), respectively. Next, we examined the relationship between treatment and the covariates (body weight, ovary weight, uterus weight, library size, liver weight, and spleen weight) and whether any of the covariates were significantly different between the treatment groups by ANOVA. Library size was not significantly associated with treatment. The other covariates (spleen, liver, uterine, and body weight) were statistically different between the treatment groups (*p* < 0.05, Table 2) for either unweighted or weighted UniFrac distances.

#### 3.2.4. Outcomes in the F1 Pups

**Figure 10:**
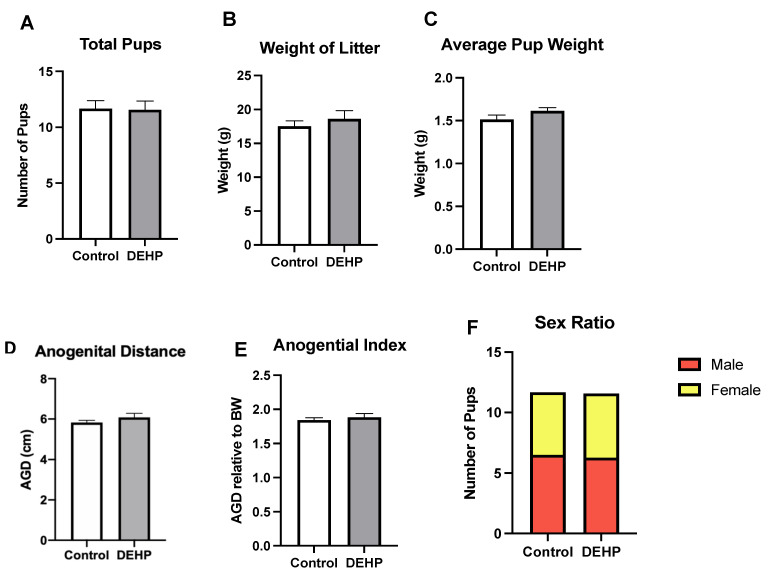
Birth and reproductive health outcomes in pups. The total number of pups (**A**), litter weight of live pups (**B**), average pup weight (**C**), anogenital distance (**D**), anogenital index (**E**), and sex ratio (**F**) are also reported below. The data presented in (**A**–**E**) are represented as mean ± standard error of the mean (SEM), *n* = 6/treatment group.

Pups were also monitored to determine whether DEHP exposure impacted their litter size, litter weight, sex ratio, and mortality. DEHP exposure did not significantly affect the litter size, litter weight, average weight of the pups, and sex ratio (Figure 10) compared with the control. Mortality rate was not significantly different between the control and DEHP treatment group. In the control group, two males and one female pup were discovered dead. Similarly, the DEHP treatment group had two dead male pups (no female pups found dead).

## 4. Discussion

Environmental chemicals, including phthalates, have been shown to alter the gut microbiome and impact reproductive outcomes in females [39]. Although DEHP impacts the gut microbiome and reproductive outcomes, studies have not examined whether DEHP-induced reproductive outcomes stem from microbiome shifts. We focused on pregnant dams because pregnant women are exposed to high levels of phthalates, and phthalates are known to be associated with undesirable pregnancy outcomes [40,41]. Specifically, the purpose of this study was to characterize the effects of DEHP exposure on the cecal microbiome in pregnant dams and determine whether the DEHP-induced microbiome shifts impact fertility and birth outcomes.

Our study revealed that exposure to DEHP during pregnancy alters the cecal microbial population in mice. Several previous studies also showed that phthalate exposure (DEHP, mono-ethylhexyl phthalate, or diethyl phthalate) modified the gut microbiome [42,43,44], and these changes were associated with obesity [43] and neurodevelopmental disorders [42]. One study showed that phthalate exposure was linked to dysbiosis in newborns, as the newborns exposed to higher levels of DEHP had significantly altered immune responses to vaccinations later in life compared with controls [45]. Another study completed in male Sprague-Dawley rats showed that DEHP exposure increased the Firmicutes/Bacteroidetes (F/B) ratio and the relative abundance in Proteobacteria in fecal samples [46]. In addition, subacute exposure to DEHP in female pubertal mice (C57Bl/6) also showed a higher alpha diversity compared with the control [42]. Wang et al. also showed increased richness in cecal alpha diversity (Chao1) in DEHP-exposed male SD rats compared with the control. In agreement with a previous study by Wang et al., the current study shows increased alpha diversity using the same alpha diversity metric in DEHP-exposed dams, despite differences in species/strain (CD-1 mouse vs. SD rat), sex (female vs. male), age (gestational vs. peripubertal), dose (20 µg/kg DEHP vs. 1000 and 3000 mg/kg DEHP), exposure time (10 days vs. 30 days), and the suspension medium used to dissolve DEHP (oil vs. water).

In the current study, DEHP exposure significantly decreased the F/B ratio compared with the control. Although different phthalates, species, and exposure time periods were used, one study in female rats also showed that phthalate (DEP) exposure decreased the F/B ratio compared with the control [44]. In contrast to these results, MEHP exposure in male mice increased the relative abundance of Firmicutes [43]. The F/B ratio was used as a marker of obesity, such that obese individuals have an increased F/B ratio, whereas normal and lean individuals have a decreased F/B ratio. By this logic, it would make sense that the obesogenic effect of DEHP is partly mediated by the gut microbiota and its microbiome. However, the F/B ratio is a high-level taxonomic index, so it is not a reliable measure of obesity or any treatment effects. Furthermore, studies in obese and lean twins have shown similar microbiota diversity [47,48].

The present study also showed that DEHP exposure during pregnancy significantly decreased the phylum Deferribacteres. The family and genus under the phylum Deferribacteres include *Deferribacteraceae* and *Mucispirillum*, respectively. Correspondingly, DEHP exposure in male mice also significantly decreased the relative abundance of *Mucispirillum* compared with the control [49]. *Mucispirillum* sp. plays a role in the murine intestinal immune microenvironment through its presence in mucus-producing goblets cells [50]. *Mucispirillum* sp. also contains systems for scavenging reactive oxygen species [50]. Its relative abundance is associated with intestinal inflammation. Excess mucus is produced in response to inflammation, so it is possible that the increased relative abundance of *Mucispirillum* helps with immune response by triggering inflammation. The decrease in *Mucispirillum* sp. in DEHP-exposed dams could be an indication of a decreased immune response in DEHP-exposed dams compared with the control. As *Mucispirillum* is associated with goblet cells, it is also possible that the DEHP exposure decreases mucus production in the gut. As a result, pathogens can pass through the intestinal barrier and induce inflammation. One study revealed that *Mucispirillum* evaded T-independent responses (which do not require assistance from helper T cells), but elicited T-dependent (which require assistance from helper T cells) immunoglobin A (IgA) [51]. Overall, *Mucispirillum* sp. seems to play a role in the intestinal immune microenvironment, but it is difficult to interpret whether *Mucispirillum* sp. would increase or decrease DEHP-induced inflammation, and subsequent research that examines this effect of DEHP on this genus in the murine GIT would add to our understanding of the effect of phthalates on the general physiology of the murine model, including reproductive physiology.

The family *Muribaculaceae* (phylum Bacteroidetes), previously known as S24-7, are dominant in mouse gut microbiota; however, little is known about them as their description, ecology, and diversity were only validated in 2019 [52]. Although limited information exists, studies suggest *Muribaculaceae* as a potential mucus degrader [52,53]. This suggests that *Muribaculaceae* may influence the intestinal immune microenvironment. A significant change was also determined in the relative abundance of *Lachnospiraceae* (phylum Firmicutes) in DEHP-exposed mice versus control mice. Like *Muribaculaceae*, *Lachnospiraceae* play a role in the intestinal immune microenvironment. Studies suggest that this taxon strengthens intestinal barriers by upregulating tight junctions, producing mucins, and downregulating pro-inflammatory cytokines [54]. However, different taxa of *Lachnospiraceae* play different roles such as producing and/or metabolizing short-chain fatty acids (SCFA), *p*-cresol, indoles, and flavonoids. Some of these metabolites like SCFA and flavonoids play a supporting role in the immune system. *Lachnospiraceae* have been shown to promote health, but some taxa have also been associated with disease [54]. Therefore, the significance of the observed changes in the *Lachnospiraceae* taxa will require more investigation.

The current study showed that DEHP exposure resulted in significant changes in several bacteria in mice. Whether these determined microbiome changes in humans are similar to the mouse model used in the current study requires further investigation as the current data cannot easily be extrapolated to humans as mice and humans are different organisms/models. The microbiome is an ecosystem that is constantly adapting and undergoing change and will vary based on model, age, lifestyle, location, sex, and diet. For example, humans have a small cecum that is barely separate from the colon and is of minor importance, whereas mice have a large cecum (3–4 cm in length) for microbial fermentation [55]. Because of the physical and functional differences, the cecal microbiome in mice is significantly different from the cecal microbiome in humans.

Metagenomic data showed that DEHP treatment alters flagellar assembly and bacterial chemotaxis pathways. Some Bacteroidetes, Firmicutes, and Proteobacteria have flagella arrangements [56]. Of the various Toll-like receptors (TLRs), TLR5 specifically recognizes bacterial flagellin. Therefore, bacterial flagellin can activate TLR5 to secrete Nf-ΚB and p38 mitogen-activated protein kinases, which induce the expression of various pro-inflammatory genes [57]. A previous study conducted in our lab showed that subacute phthalate exposure increased gene expression of proinflammatory cytokines, including tumor necrosis factor (*Tnf*) and interferon-gamma (*Ifng*), compared with the control [58]. Although DEHP treatment significantly decreased the relative abundance of Firmicutes and did not significantly alter the relative abundance of Proteobacteria, these observations at the phylum level give a high-level overview of what may be happening based on which microbes are present. However, DEHP exposure significantly increased the relative abundance of Bacteroidetes compared with the control, thereby increasing the number of bacteria with flagellin to induce inflammatory responses. It is possible that species or strains within this phylum have a major impact on inducing inflammatory responses through flagellin-activated TLR5.

Another possible explanation for proinflammatory responses induced by peritrichous bacteria (i.e., bacteria with flagella around the entire cell body) is the use of their flagella to move against a chemical concentration gradient to colonize a specific area (also known as chemotaxis) and to trigger an inflammatory response. For instance, *Helicobacter pylori*, a well-studied peritrichous pathogen, uses chemotaxis to invade gastric epithelial cells and trigger the release of pro-inflammatory cytokines and chemokines [59]. Pathways in bacterial chemotaxis and flagellar assembly are enriched in DEHP-treated mice compared with the control, and these components make up a potent inflammatory milieu [60,61]. Furthermore, DEHP exposure borderline increased lipopolysaccharide (LPS) biosynthesis, which could further exacerbate inflammation in DEHP-exposed dams. Although immune factors, including LPS, were not measured in the current study, results from shotgun metagenomic sequencing showed that the LPS biosynthesis pathway was borderline dysregulated, which suggests that LPS levels may be abnormal in mice exposed to DEHP. LPS is synthesized in the cytoplasm of gram-negative bacteria and then exported to the outer leaflet of the cell wall, making LPS a major constituent of gram-negative bacteria [62,63]. Gram-negative bacteria can then release LPS to stimulate the release of inflammatory cytokines and trigger innate immune responses [64]. As demonstrated in this current study, DEHP exposure significantly increased the relative abundance of Bacteroidetes, a phylum of gram-negative bacteria, compared with the control, supporting the evidence that LPS biosynthesis from gram-negative bacteria plays a role in dysregulating immune responses in the gut.

In the present study, DEHP exposure during gestation did not impact pregnancy or pup outcomes. Similar studies have reported that phthalate exposure does not impact fertility outcomes in dams; however, fertility issues in the pups were observed in a multigenerational and transgenerational manner [65,66,67]. Specifically, DEHP exposure in pregnant CD-1 mice significantly decreased the gestational index in a multigenerational manner compared with the control [65]. However, DEHP exposure did not affect the gestational index in the first filial generation (F1), which supports the findings reported in this study. Although there were significant differences in the litter size and sex ratio in the second (F2) and third filial (F3) generation compared with the control, Rattan et al. also reported that DEHP exposure did not affect the litter size, sex ratio, and pup mortality in the F1 generation [65], which agrees with the findings in the current study. Previous studies have also shown that DEHP significantly impacted pregnancy outcomes at or above 200 µg/kg bw/day [9,10,68]. The doses used in previous studies are different than those in the current study, which only examined DEHP exposure at 20 µg/kg bw/day. In addition to different doses, different mouse models and experimental designs were used in previous studies and the current study [9,10,68]. Of note, DEHP-induced shifts in the microbiome observed in this study did not impact fertility or birth outcomes in the F1 generation.

Whether the current studies can be extrapolated to humans requires more investigation. Several studies showed that newborns exposed to higher levels of DEHP in utero had a significantly shorter pregnancy duration compared with newborns exposed to lower levels of DEHP [6,69,70]. However, human evidence is inconsistent owing to different study populations, study design limitations, recruitment, and heterogeneity of the epidemiological studies [69,70,71].

## 5. Conclusions and Future Directions

In conclusion, exposure to DEHP significantly altered the cecal microbiota as well as the cecal microbiome in pregnant dams. Many of the significant microbial changes seem to play a role in the intestinal immune system, but bacteria within the same taxa (i.e., family) can have opposing roles. Identification of functional pathways significantly altered by DEHP exposure show that DEHP could impact the whole-body system, but these functional changes need to be studied in detail to gain insight and truly understand the impact of this environmental toxicant on microbial community diversity, dynamics, and function, and thus their impact on the host. One of the major limitations of this study is the lack of information on whether the observed functional changes in the gut were apparent in the reproductive organs, such as the ovaries and uterus. The study design was also not able to include nonpregnant mice to understand whether similar microbiota changes are seen in non-pregnant mice exposed to DEHP and whether these changes are dependent on pregnancy. Therefore, future studies should focus on whether the determined functional changes from metagenomic sequencing affect metabolic and physiological conditions by examining the ovary and uterus in closer detail and including DEHP-exposed nonpregnant mice to determine whether microbiota changes are necessary for pregnancy. Overall, DEHP exposure in pregnant mice did not significantly impact the fertility and birth outcomes compared with the control. These data indicate that DEHP exposure during pregnancy shifts the cecal microbiome, but the microbiome shifts do not adversely impact fertility and birth outcomes.

## Figures and Tables

**Table 1:** Number of taxa with assigned ranks at each level.

Classification	Number of Taxa
Kingdom	178
Phylum	178
Class	178
Order	178
Family	168
Genus	66
Species	4

**Table 2:** PERMANOVA *p*-values for determining the association of treatment and/or covariates with distance matrices in pregnant mice. For the combined Trt + covariate entries (Trt + Uterus weight, Trt + Library size, Trt + Liver weight, and Trt + Spleen weight), the *p*-values are shown for Trt. Rows with any significance (*p* < 0.05) are bolded in blue, and rows with any marginal significance (*p* < 0.10) are bolded in black.

Variable	Bray–Curtis	UniFrac	Weighted UniFrac
**Trt**	**0.012**	**0.009**	**0.017**
**Body weight**	**0.452**	**0.433**	**0.082**
Ovary weight	0.483	0.461	0.585
**Uterus weight**	**0.649**	**0.057**	**0.872**
**Liver weight**	**0.291**	**0.673**	**0.046**
**Spleen weight**	**0.156**	**0.190**	**0.072**
Library size	0.477	0.624	0.108
**Trt + Body Weight**	**NA**	**NA**	**0.012**
**Trt + Uterus Weight**	**NA**	**0.007**	**NA**
**Trt + Liver Weight**	**NA**	**NA**	**0.008**
**Trt + Spleen Weight**	**NA**	**NA**	**0.016**

**Table 3:** Functional pathways examined in control and DEHP-exposed dams. Rows showing significant differences between DEHP treatment and control are bolded in blue (*p* < 0.05). *n*, number of genes in GO term. DE, number of genes in the DE set.

Pathway ID	Pathway	*n*	DE	P.DE
**path:ko02040**	**Flagellar assembly**	**30**	**24**	**4.34 × 10^−8^**
**path:ko02030**	**Bacterial chemotaxis**	**21**	**16**	**2.70 × 10^−5^**
**path:ko03060**	**Protein export**	**18**	**12**	**2.02 × 10^−3^**
**path:ko00190**	**Oxidative phosphorylation**	**45**	**23**	**3.94 × 10^−3^**
**path:ko00720**	**Carbon fixation pathways in prokaryotes**	**53**	**26**	**4.60 × 10^−3^**
**path:ko02024**	**Quorum sensing**	**86**	**38**	**6.89 × 10^−3^**
**path:ko03440**	**Homologous recombination**	**21**	**12**	**1.19 × 10^−2^**
**path:ko00340**	**Histidine metabolism**	**20**	**11**	**2.26 × 10^−2^**
**path:ko01100**	**Metabolic pathways**	**898**	**304**	**2.45 × 10^−2^**
**path:ko00130**	**Ubiquinone and other terpenoid-quinone biosynthesis**	**18**	**10**	**2.71 × 10^−2^**
**path:ko00121**	**Secondary bile acid biosynthesis**	**3**	**3**	**3.02 × 10^−2^**
**path:ko01502**	**Vancomycin resistance**	**16**	**9**	**3.25 × 10^−2^**
**path:ko01200**	**Carbon metabolism**	**152**	**58**	**3.65 × 10^−2^**
**path:ko00020**	**Citrate cycle (TCA cycle)**	**37**	**17**	**4.11 × 10^−2^**
path:ko00061	Fatty acid biosynthesis	17	9	5.04 × 10^−2^
path:ko00540	Lipopolysaccharide biosynthesis	25	12	5.75 × 10^−2^
path:ko03430	Mismatch repair	20	10	6.05 × 10^−2^
path:ko00250	Alanine, aspartate, and glutamate metabolism	36	16	6.37 × 10^−2^
path:ko01210	2-Oxocarboxylic acid metabolism	28	13	6.43 × 10^−2^
path:ko01110	Biosynthesis of secondary metabolites	350	122	6.64 × 10^−2^
path:ko04142	Lysosome	8	5	6.73 × 10^−2^
path:ko00780	Biotin metabolism	13	7	7.48 × 10^−2^
path:ko00290	Valine, leucine and isoleucine biosynthesis	13	7	7.48 × 10^−2^
path:ko00531	Glycosaminoglycan degradation	6	4	7.99 × 10^−2^
path:ko00401	Novobiocin biosynthesis	6	4	7.99 × 10^−2^
path:ko03018	RNA degradation	16	8	9.01 x 10^-2^
path:ko00550	Peptidoglycan biosynthesis	32	14	9.06 × 10^−2^
path:ko03013	RNA transport	4	3	9.27 × 10^−2^
path:ko04214	Apoptosis—fly	2	2	9.71 × 10^−2^
path:ko00332	Carbapenem biosynthesis	2	2	9.71 × 10^−2^

## Data Availability

The data are available from the investigators upon reasonable request.
